# The differences of serum lipid profiles between primary aldosteronism and essential hypertension: a meta-analysis and systematic review

**DOI:** 10.1186/s12902-022-01135-y

**Published:** 2022-08-31

**Authors:** Worapaka Manosroi, Pitchaporn Phudphong, Pichitchai Atthakomol, Mattabhorn Phimphilai

**Affiliations:** 1 Department of Internal Medicine, Faculty of Medicine, Chiang Mai University, 110 Intrawarorot Road Soi 2, Si Phum, Amphoe Mueang Chiang Mai, Chiang Mai, 50200 Thailand; 2 Orthopaedics Department, Faculty of Medicine, Chiang Mai University, Muang Chiang Mai, Chiang Mai, Thailand; 3 Clinical Epidemiology and Clinical Statistic Center, Faculty of Medicine, Chiang Mai University, Chiang Mai, Thailand

**Keywords:** Primary aldosteronism, Essential hypertension, Lipid profiles, Triglycerides, Cholesterol, HDL, LDL

## Abstract

**Background:**

The data on lipid profile differences between primary aldosteronism (PA) and essential hypertension (EH) patients are inconsistent and inconclusive. Most studies reported lower levels of lipid profiles in PA than in EH. This meta-analysis aimed to explore differences in serum lipid profiles including triglyceride (TG), total cholesterol (TC), LDL and HDL levels in PA patients and EH patients.

**Methods:**

A search of published studies was performed using *PubMed*, *Embase* and *Scopus* databases from their inception through August 2022. Thirty studies involving 11,175 patients were identified. Inclusion criteria included 1) observational studies which contained data on any of the lipid profiles of interest (TG, TC, LDL and HDL) which could be acquired from baseline data or the outcomes, 2) data which should be compared between adult PA and EH patients and 3) the use of appropriate methods to diagnose PA. Standardized mean difference (SMD) with a 95% confidence interval (95% CI) was calculated to assess effect size by using STATA program version 15.0. Risk of bias was assessed by Joanna Briggs Institute (JBI) Critical Appraisal Tools for cross-sectional, cohort and case-control studies.

**Results:**

Levels of the lipid parameters TG (SMD − 0.16 mmol/L; 95%CI (− 0.25, − 0.07)), TC (SMD − 0.30 mmol/L; 95%CI (− 0.41, − 0.19)) and LDL (SMD − 0.17 mmol/L; 95%CI (− 0.27, − 0.08)) were significantly lower in PA than in EH patients. There was no statistically significant difference in HDL between PA and EH patients (SMD − 0.08 mmol/L; 96%CI (− 0.23,0.07)). High levels of heterogeneity for TG, TC, HDL and LDL were observed in all studies. Risk of bias among the studies was low to moderate.

**Conclusion:**

Lower levels of TG, TC and LDL were observed in PA than in EH patients. Further study should be conducted to address the underlying mechanisms of lipid alteration in PA.

**Supplementary Information:**

The online version contains supplementary material available at 10.1186/s12902-022-01135-y.

## Introduction

Primary aldosteronism (PA), a syndrome described by autonomous excess aldosterone production, is recognized as a common form of secondary hypertension. The reported prevalence of PA ranges from 5 to 20% depending on the study, patient selection and the severity of hypertension [1]. Inappropriate aldosterone secretion in PA patients causes intravascular volume expansion, sodium retention, suppression of plasma renin level, hypertension and increased potassium secretion which can lead to hypokalemia [2]. Patients with PA were reported to be at greater risk of multiple end-organ damage, including atherosclerotic cardiovascular disease, cerebrovascular disease and renal sequelae which can cause more damage than the degree of hypertension itself, compared to essential hypertension (EH) patients [3]. Apart from the effect on intravascular volume and salt regulation, increased aldosterone levels can cause oxidative stress and proinflammatory effects on vascular walls. These effects are key mechanisms in the pathogenesis of atherosclerosis and can lead to multiple end organ damage [4]. In addition, PA has been reported to be related to metabolic syndrome and insulin resistance [5]. Also, a previous meta-analysis demonstrated a higher risk of glycemic abnormalities in PA than in EH patients. Due to the strong association of PA with atherosclerosis and metabolic syndrome, potential alteration in serum lipid profiles may be observed in some PA patients.

In normal populations without PA, multiple studies have found a positive correlation between low-density lipoprotein cholesterol (LDL), non-high-density lipoprotein cholesterol (non-HDL) and plasma aldosterone concentration, while a negative correlation with high-density lipoprotein cholesterol (HDL) has been observed [6, 7]. Deterioration of serum lipid profiles in PA patients after successful treatment by either adrenalectomy or mineralocorticoid antagonist were also observed despite an unchanged body mass index (BMI), improvement in fasting blood glucose and blood pressure [8]. Apart from PA, significant differences in lipid profiles compared to the control group were reported in some special populations, e.g., pregnancy-induced hypertension patients [9].

Dyslipidemia is a major contributor to atherosclerotic cardiovascular disease and cerebrovascular disease. According to the relationship between PA and atherosclerosis, higher probability of alteration of lipid profiles might be observed in PA than in EH patients. However, data on differences in alterations of serum lipid profiles in PA and EH patients are still inconclusive. Decreased levels of HDL in PA compared to EH has been demonstrated in some studies [10–12] which could be an explanatory mechanism related to the higher risk of atherosclerosis in PA than in EH. Interestingly, plasma total cholesterol (TC), triglycerides (TG), HDL and LDL have all been demonstrated to be significantly lower in PA than in EH in multiple studies [12, 13]. Also, no difference between the profiles of these lipids between PA and EH patients has been observed in several studies [14–16]. Because of the conflicting and inconclusive results related to serum lipid profiles in PA patients, this meta-analysis was performed to systematically elucidate differences in serum lipid profiles including TG, TC, LDL and HDL levels in PA compared to EH patients.

## Materials and methods

### Search strategy and selection criteria

Our meta-analysis adhered the Preferred Reporting Items for Systematic Reviews and Meta-analyses (PRISMA) guidelines [17]. The prespecified protocol was registered in PROSPERO (CRD42021287330). The comprehensive search from three databases including *PubMed/Medline*, *Scopus* and *Embase*, was conducted from their inception to August 2022. The terms “Hyperaldosteronism OR aldosteronism OR primary aldosteronism” AND “lipid OR lipids OR cholesterol OR triglyceride OR low-density lipoprotein OR high density lipoprotein OR metabolic syndrome OR metabolic OR apolipoprotein A OR apolipoprotein B” were used as the keywords. Medical subject heading (MeSH) terms employed in the *PubMed* search and *Emtree* were employed in *Embase*. (For details, please see Supplementary Appendix.) To identify additional studies, a manual search was also performed of the reference lists from the included studies and from non-included reviews. Initial screening of abstracts and titles and duplicate article removal were performed using Rayyan, a web-based program (Rayyan Systems Inc., Cambridge, MA, USA) [18].

Two researchers (WM, PP) independently performed the searches, screened for titles and abstracts and reviewed full-text papers for eligibility criteria. Relevant studies from skimming titles and abstracts were retrieved for full text and were screened for eligibility criteria. Researchers (WM, PP) evaluated the methodological quality of the included studies and performed the data extraction independently. Any disagreements were debated and resolved by consensus with the third author (PA).

Eligibility criteria included the following: 1) comparative non-randomized observational studies (prospective cohort, retrospective cohort, case-control or cross-sectional study) which contained lipid profiles of at least one of the following TG, TC, LDL or HDL. The lipid profile data could be retrieved from primary outcome, baseline characteristics or baseline investigations.; 2) data should be compared between PA and EH adult patients (age ≥ 18 years).; and 3) use of appropriate methods recommended by standard guidelines for diagnosing and confirming PA [2]. Exclusion criteria were studies published in non-English language, review articles, case reports, abstracts, and animal studies. Articles including vulnerable populations such as pregnancy were also excluded. In the case of multiple studies involving the same cohort, only the study with highest number of patients was selected.

### Data extraction

Two authors (WM, PP) conducted the data extraction independently. The demographic variables extracted from each study were: 1) study characteristics including the name of the first author, year of publication, country in which conducted, study design, whether the study was demographically matched between PA and EH, whether it excluded statin users, and the number of PA and EH patients; 2) patient characteristics and potential confounding factors, i.e., means and standard deviations (SD) of age, percentage of the predominant sex, predominant ethnicity, mean BMI and mean fasting plasma glucose (FPG); 3) outcome variables, i.e., mean and SD of serum lipid profiles including TG, TC, LDL and HDL in each group of PA and EH patients. Serum lipid profiles were retrieved from baseline characteristics if the endpoint of the studies were not aimed for differences of lipid levels. All the mean values were converted and presented as SI units.

### Data synthesis

STATA program version 15.0 was used to perform the data analysis. Also, κ statistic to determine the level of agreement between reviewers for study selection was performed. A κ value of > 0.8 indicates excellent agreement [19]. Standardized mean difference (SMD) with a 95% confidence interval (95% CI) was analyzed to assess effect size. To estimate the statistical heterogeneity among the studies, the I^2^ statistic was calculated. I^2^ values of 25, 50 and 75% indicated low, medium and high heterogeneity, respectively [20]. We used the random-effect modeling used for the data analysis. The statistically significant level for this meta-analysis was set at *p* < 0.05. Publication bias was evaluated using funnel plots and Egger’s linear regression tests. The asymmetry of funnel plot demonstrated publication bias. A *p*-value < 0.05 indicated statistically significant publication bias for Egger’s regression.

Subgroup analysis was also performed to demonstrate the effect of age, ethnicity, BMI, FPG, demographic data matching and statin use. We performed subgroup analysis by age since some publications had reported differences in serum lipid profiles between older and younger populations [21]. Older and younger groups were categorized as mean age above and below 50 years as it has been reported that at the age of 50 years, serum lipid levels may reach their peak levels [22]. Sex was stratified based on male-predominant studies where the study population included ≥50% males. Regarding ethnicity, comparison was performed between Asians and non-Asians as a study has presented a difference in serum lipid levels between Asians and non-Asians, even in individuals with normal BMI [23]. In Asians, BMI was separated into means above and below 25 kg/m^2^ and in non-Asians into means above and below 30 kg/m^2^ [24]. BMI above and below the cut-off was used to define obese and non-obese patients, respectively. Subgroup analysis by BMI was performed as a study reported a correlation between BMI and alteration in lipid profiles [25]. FPG was categorized based on impaired fasting blood glucose criteria which was the mean blood glucose above and below 5.6 mmol/L, based on evidence that high blood glucose values are linked with significant lipid derangement of profiles [26]. Subgroup analysis was also conducted by dividing studies into those with and without data matching in order to adjust the confounders. Studies with at least one matched variable (sex, age, BMI, blood pressure or duration of hypertension) were classified to subgroups with demographic data matching. Subgroup analysis by statin use was categorized to no statin use and statin use/unknown groups. Only the outcome of TG was performed because there was only one study in the no statin use group for TC, LDL and HDL outcomes. As there were various study types included in this meta-analysis, a sensitivity analysis was also performed by removing cross-sectional studies, and only case-control and cohort studies were analyzed.

### Risk of bias assessment

Assessment of the risk of bias was performed independently by two authors (WM and PP) using the Joanna Briggs Institute (JBI) Critical Appraisal Tools for cross-sectional, case-control and cohort studies [27].

### Certainty of the evidence

Two authors (WM and PP) assessed the quality of the evidence independently by using Grading of Recommendation, Assessment Development and Evaluation (GRADE) methodology. The details of GRADE are available elsewhere [28]. Any conflicts were resolved by the third author (PA).

## Results

**Fig. 1:**
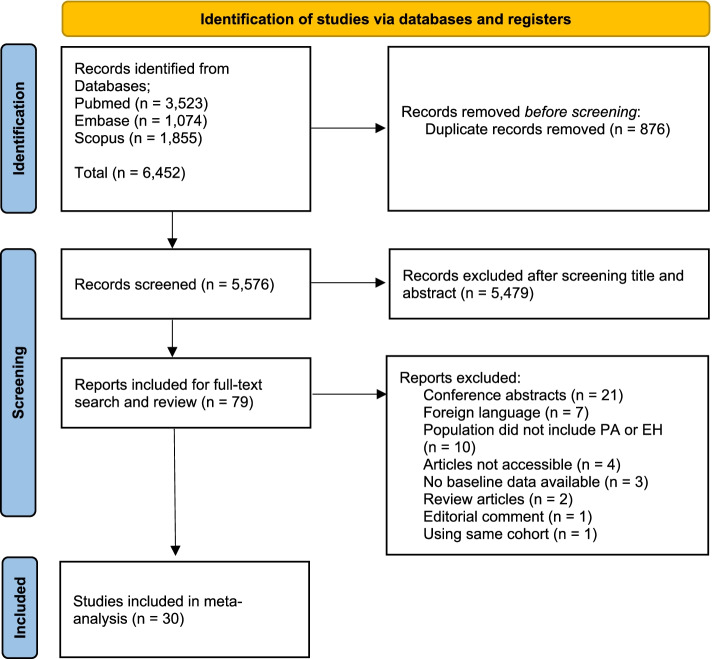
Flowchart of study selection process

The database searches yielded 6452 articles, including 3523 from *PubMed*, 1074 from *Embase* and 1855 from *Scopus*. From that total, 876 duplicates were removed. The titles and abstracts of 5576 articles were screened resulting in the exclusion of 5479 articles as irrelevant to our aim. The full texts of the remaining 79 articles were retrieved and reviewed resulting in the exclusion of 49 articles due to various reasons. A total of 30 studies were finally included [10–15, 29–52]. There was excellent inter-reviewer agreement (κ = 0.82; 95% CI, 0.71–0.92). The study selection process is shown in Fig. 1.

### Study characteristics

Characteristics of the included studies are summarized in Table 1. The 30 studies included in this meta-analysis present the differences in lipid profiles in PA compared to and EH patients. Most of the studies were cross-sectional and most were conducted in non-Asian populations. All studies provided data on sex, age, ethnicity, and BMI and 23 studies provided data on FPG [11, 14, 15, 29–37, 39–42, 45–51]. Only one study excluded patients who used statins [10]. In one study, statins were withdrawn for 4 weeks prior to lipid profile evaluation [47], while five studies stated that statins were continued during the study [12, 30, 33, 40, 51]. The remaining studies did not mention this issue. One study excluded patients with severe hyperlipidemia [34]. All but one [29] of the studies provided data on TG levels, 27 included data on TC [10–15, 29, 31, 33–50, 52] and HDL levels [10–15, 29–36, 38–41, 43, 45–52] and 25 provided LDL levels [10–15, 29, 31, 33–36, 38–41, 43, 45–52].

**Table 1:** Baseline characteristics of the included studies

Author	Year	Number of patients (PA/EH)	Study design	Male (%)	Mean age ± SD (years)	Mean BMI ± SD (kg/m2)	Mean fasting blood glucose ± SD (mmol/L)	Ethnic	Demographic data matching	Statin use	Risj of bias by JBI
Strauch [29]	2006	36/28	Cross-sectional	50	50.7 ± 10.2	27.9 ± 4.6	4.9 ± 0.59	Non-Asian	No	N/A	Moderate
Fallo [30]	2006	85/381	Cross-sectional	57.2	53.4 ± 12.4	27.8 ± 4.5	5.4 ± 1.14	Non-Asian	No	Yes	Low
Catena [31]	2006	47/274	Cohort	71	53 ± 12	28.4 ± 2.9	4.9 ± 1	Non-Asian	Yes	N/A	Moderate
Ronconi [32]	2009	89/164	Cross-sectional	52.2	51 ± 11	27.4 ± 4.3	5.3 ± 1.1	Non-Asian	Yes	N/A	Low
Matrozova [14]	2009	458/1346	Cross-sectional	66.2	51.7 ± 10.4	27.3 ± 4.6	5.5 ± 0.8	Non-Asian	Yes	N/A	Low
Somloova [33]	2010	99/90	Cross-sectional	54.2	49.9 ± 10.1	28.7 ± 4.6	5.3 ± 1.2	Non-Asian	Yes	Yes	Low
Fallo [34]	2010	40/40	Cross-sectional	65	52 ± 9	26.7 ± 2.8	5.2 ± 0.8	Non-Asian	Yes	Severe hyperlipidemia excluded	Low
Stehr [35]	2010	30/70	Cross-sectional	28	54.7 ± 10.8	30.1 ± 5.4	4.9 ± 0.8	Non-Asian	Yes	N/A	Low
Iacobellis [36]	2012	75/192	Cross-sectional	55	54.9 ± 12.4	26.9 ± 3.6	5.9 ± 0.3	Non-Asian	No	N/A	Low
Reincke [13]	2012	300/600	Case-control	61	50 ± 0	28 ± 03.9	N/A	Non-Asian	Yes	N/A	Low
Savard [37]	2013	457/1273	Cross-sectional	65.6	51.2 ± 10.3	27.9 ± 5.0	5.8 ± 1.6	Non-Asian	Yes	N/A	Low
Prejbisz [38]	2013	32/172	Cross-sectional	60.3	48.4 ± 7.7	30.1 ± 4.7	N/A	Non-Asian	No	N/A	Moderate
Liu [39]	2014	50/51	Cross-sectional	64.3	41.8 ± 11.5	22.5 ± 3.2	4.8 ± 0.5	Asian	Yes	N/A	Low
Turchi [11]	2014	102/132	Cohort	49.5	52.1 ± 10.5	27.2 ± 3.6	5.7 ± 1.4	Non-Asian	Yes	N/A	Moderate
Choudhary [40]	2015	130/130	Cross-sectional	65	52.9 ± 16.9	29.9 ± 9.7	6.4 ± 2.7	Non-Asian	Yes	Yes	Moderate
Yang [41]	2016	100/100	Cross-sectional	58	50 ± 12	25.5 ± 3.3	5.4 ± 1.2	Asian	Yes	N/A	Low
Watanabe [42]	2016	32/21	Cross-sectional	35.8	56.0 ± 12.3	23.5 ± 4.5	5.3 ± 1.1	Asian	Yes	N/A	Low
Monticone [15]	2017	99/1573	Cross-sectional	56.5	46.2 ± 8.9	26.1 ± 4.3	5.2 ± 1.1	Non-Asian	No	N/A	Low
Berends [12]	2018	20/644	Cross-sectional	59.3	58.7 ± 11.3	27.8 ± 4.4	N/A	Non-Asian	Yes	Yes	Low
Li [43]	2018	27/27	Cross-sectional	48.1	43.8 ± 11.9	24.1 ± 3.7	N/A	Asian	No	N/A	Low
Er [44]	2019	100/41	Cross-sectional	41.8	50.4 ± 12.1	24.9 ± 3.7	N/A	Asian	Yes	N/A	Low
Vujacik [45]	2020	40/40	Cross-sectional	35	52.8 ± 11.8	28.1 ± 4.5	5.28 ± 1.1	Non-Asian	No	N/A	Low
Manosroi [46]	2020	41/38	Cross-sectional	35.4	38.4 ± 14.5	29.6 ± 6.8	5.42 ± 1.2	Asian	No	N/A	Low
Zhang [47]	2020	109/109	Cross-sectional	40.4	45 ± 9.6	24.1 ± 3.8	5.42 ± 1.3	Asian	Yes	Withdrawn 4 weeks before analysis	Low
Hu [48]	2020	49/207	Cross-sectional	58.6	49.9 ± 11.8	26.4 ± 3.7	7.7 ± 2.7	Asian	Yes	N/A	Low
Caprino [49]	2020	32/78	Cross-sectional	55	58.1 ± 11.2	29 ± 4.5	5.3 ± 0.7	Non-Asian	No	N/A	Low
Sang [50]	2021	36/31	Cross-sectional	47	50.8 ± 12.4	25.6 ± 4.0	5.1 ± 0.2	Asian	No	N/A	Moderate
Moon [10]	2021	80/80	Cross-sectional	52.5	51.7 ± 12.9	25.3 ± 3.6	N/A	Asian	Yes	No	Low
Fernández-Argüeso [51]	2021	50/50	Case-control	57	59.3 ± 24.5	30.6 ± 11.9	5.5 ± 3.4	Non-Asian	Yes	Yes	Low
Huang [52]	2021	174/174	Case-control	48.3	46.2 ± 12.6	24.7 ± 3.9	N/A	Asian	Yes	N/A	Low

*N/A* not available, *PA* Primary aldosteronism, *EH* Essential hypertension, *JBI* Joanna Briggs Institute (JBI) Critical Appraisal Tools for cross-sectional study

### Risk of bias in the studies

Risk of bias was evaluated using JBI tools for cross-sectional, case-control and cohort studies (Table 1). Details of the scores in each study are shown in the Supplementary Appendix. In terms of risk of bias, the majority of the studies (24 of 30) evidenced high quality with a low risk of bias [10, 12–15, 30, 32–37, 39, 41–49, 51, 52]. Four cross-sectional studies demonstrated moderate quality with a moderate risk of bias as important confounders had not been appropriately controlled in those studies [29, 38, 40, 50]. Two cohort studies with moderate risk of bias were not assessed regarding the outcomes issues as measured outcomes were not relevant to the primary objectives of the current study [11, 31].

### Results of syntheses

**Fig. 2:**
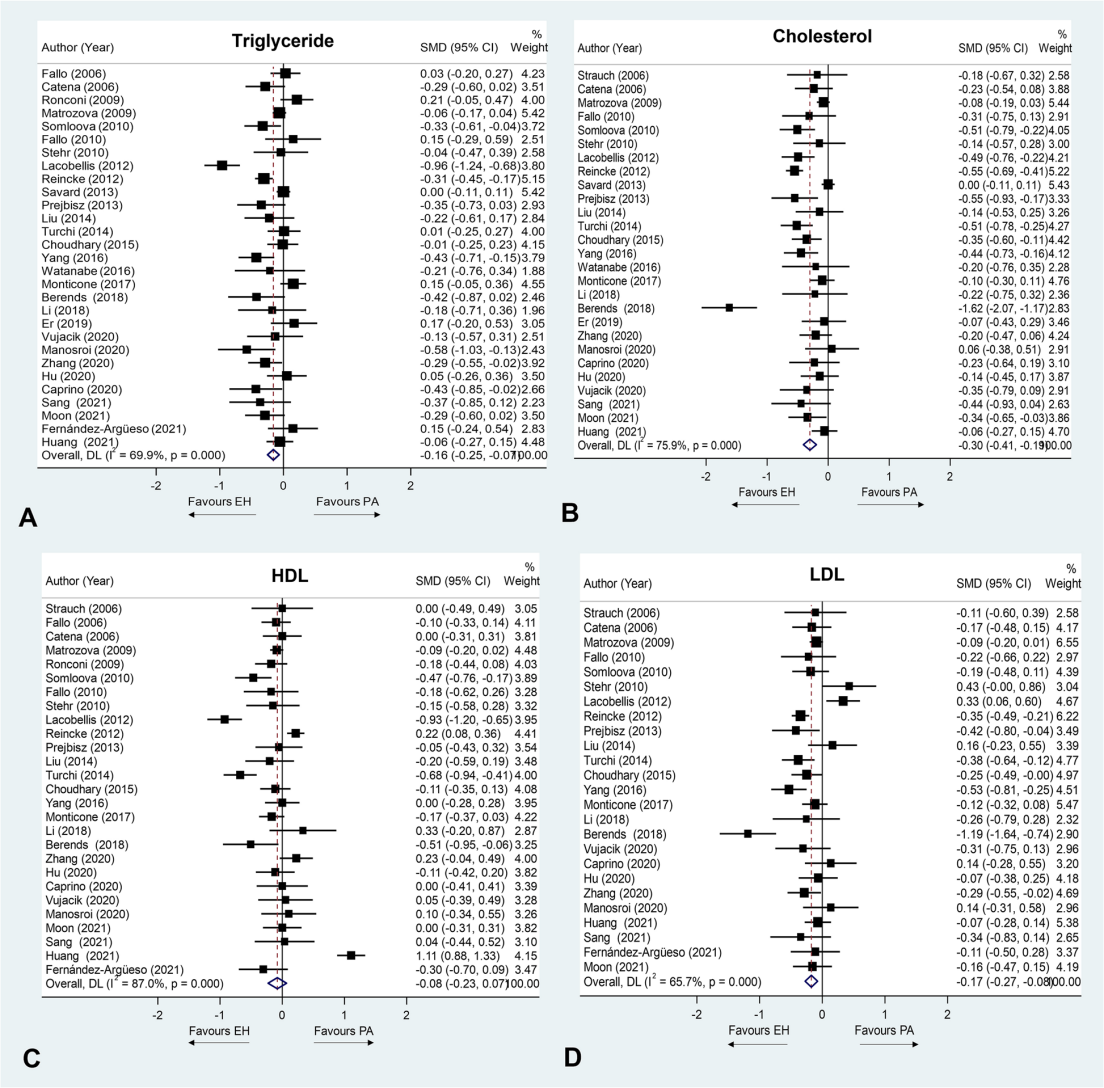
Forest plot of the mean difference of triglyceride (**A**), cholesterol (**B**), HDL (**C**) and LDL (**D**) levels between primary aldosteronism and essential hypertension patients

A total of 11,175 patients from 30 studies were included in this meta-analysis. Forest plots of each lipid profile are shown in Fig. 2. Subgroup analysis results are showed in Table 2 and figures are shown in Supplementary Appendix. Data of publication bias are shown in Supplementary Appendix.

**Table 2:** Subgroup analysis for assessment of lipid profiles between primary aldosteronism and essential hypertension patients

Subgroup	Number of studies	SMD (95%CI)	I^**2**^ (%)	I^**2**^ ***p***-value
**Triglyceride**				
Mean age				
- < 50 years	11	− 0.21 (− 0.34, − 0.07)	60.7	0.005
- ≥ 50 years	18	− 0.13 (− 0.25, 0.00)	72.4	< 0.001
Ethnicity				
-Asian-predominant	11	− 0.20 (− 0.33, − 0.07)	31.5	0.147
-Non-Asian- predominant	18	−0.14 (− 0.26, − 0.02)	77.6	< 0.001
Mean BMI				
-Non-obese	19	−0.16 (− 0.27, − 0.05)	75.8	< 0.001
-Obese	10	−0.16 (− 0.31, − 0.00)	50.1	0.035
Mean plasma glucose				
-Normal	18	−0.12 (− 0.23, − 0.02)	52.2	0.005
-Impaired fasting glucose	6	−0.19 (− 0.47, 0.08)	88.5	< 0.001
Demographic data matching				
-Unmatched	9	−0.31 (− 0.59, − 0.02)	83.7	< 0.001
-Matched	20	−0.11 (− 0.19, − 0.03)	52.5	0.003
Statin use				
-Non-statin	2	−0.29 (− 0.49, − 0.09)	0	0.987
-Unknown	27	−0.15 (− 0.25, − 0.05)	71.1	< 0.001
**Cholesterol**				
Mean age				
- < 50 years	11	−0.27 (− 0.42, − 0.12)	67.1	0.001
- ≥ 50 years	16	−0.33 (− 0.48, − 0.18)	78.5	< 0.001
Ethnicity				
-Asian-predominant	11	−0.19 (− 0.29, − 0.09)	0	0.584
-Non-Asian- predominant	16	−0.37 (− 0.52, − 0.21)	84.8	< 0.001
Mean BMI				
-Non-obese	18	−0.31 (− 0.45, − 0.17)	82.6	< 0.001
-Obese	9	−0.29 (− 0.41, − 0.17)	10.0	0.352
Mean plasma glucose				
-Normal	16	−0.18 (− 0.26, − 0.10)	10.8	0.330
-Impaired fasting glucose	6	−0.29 (0.51, − 0.08)	80.0	< 0.001
Demographic data matching				
-Unmatched	9	−0.27 (− 0.42, − 0.13)	23.2	0.237
-Matched	18	−0.31 (− 0.45, − 0.17)	82.5	< 0.001
**HDL**				
Mean age				
- < 50 years	11	0.09 (−0.17, 0.36)	90.5	< 0.001
- ≥ 50 years	16	−0.21 (− 0.35, − 0.07)	71.6	< 0.001
Ethnicity				
-Asian-predominant	9	0.17 (−0.16, 0.57)	89.0	< 0.001
-Non-Asian- predominant	18	−0.20 (− 0.34, − 0.06)	79.6	< 0.001
Mean BMI				
-Non-obese	18	−0.09 (− 0.29, 0.12)	91.3	< 0.001
-Obese	9	−0.07 (− 0.18, 0.04)	0	0.939
Mean plasma glucose				
-Normal	18	−0.01 (− 0.19, 0.17)	85.5	< 0.001
-Impaired fasting glucose	5	−0.37 (− 0.73, − 0.01)	87.8	< 0.001
Demographic data matching				
-Unmatched	9	−0.10 (− 0.33, 0.12)	75.5	< 0.001
-Matched	18	−0.07 (− 0.26, 0.12)	89.6	< 0.001
**LDL**				
Mean age				
- < 50 years	11	−0.20 (− 0.32, − 0.08)	48.3	0.036
- ≥ 50 years	14	−0.16 (− 0.32, − 0.00)	72.5	< 0.001
Ethnicity				
-Asian-predominant	9	−0.17 (− 0.31, − 0.02)	41.2	0.093
-Non-Asian- predominant	16	−0.18 (− 0.31, − 0.05)	73.4	< 0.001
Mean BMI				
-Non-obese	16	−0.18 (− 0.30, − 0.05)	70.7	< 0.001
-Obese	9	−0.17 (− 0.34, 0.01)	56.7	0.018
Mean plasma glucose				
-Normal	16	−0.12 (− 0.22, − 0.03)	35.2	0.081
-Impaired fasting glucose	5	−0.11 (− 0.35, 0.14)	75.2	0.003
Demographic data matching				
-Unmatched	9	−0.08 (− 0.26, 0.10)	52.3	0.033
-Matched	16	−0.22 (− 0.33, − 0.10)	69	< 0.001

*SMD* standardized mean difference, *CI* Confidence interval, *BMI* Body mass index

#### Triglyceride

Patients with PA showed significantly lower SMD than in EH patients (SMD − 0.16 mmol/L; 95%CI (− 0.25, − 0.07)). For subgroup analysis, significantly lower TG levels were observed in PA compared to EH patients in studies of patients with normal FPG (SMD − 0.12 mmol/L; 95%CI (− 0.23, − 0.02), but not for patients with mean FPG in the impaired range. Significantly lower TG levels were observed in PA compared to EH patients in the subgroups of age, ethnicity, BMI, demographic data matching and statin use. In TG studies, moderate heterogeneity (I^2^ = 69.9, *p* < 0.001) was observed. Ethnicity was found to be the cause of heterogeneity. Studies performed in groups where Asians predominated were more homogeneous than the those done in groups that were predominately non-Asians. Egger’s regression test did not reveal any publication bias for TG with *p* = 0.191.

#### Total cholesterol

PA patients demonstrated significantly lower cholesterol levels than EH patients (SMD − 0.30 mmol/L; 95%CI (− 0.41, − 0.19)). For subgroup analysis, as in the general findings, PA patients generally had significantly lower TC than EH patients for the subgroups of age, ethnicity, BMI, FPG, and demographic data matching. There was high heterogeneity among the studies in TC and HDL levels (I^2^ = 75.9%, *p* < 0.001)). Ethnicity distribution, mean BMI and FPG and whether the demographic data had been matched were sources of heterogeneity The studies conducted mainly in Asians and those with unmatched demographic data showed more homogeneous patterns than other subgroups. Also, studies of predominantly obese patients and those with impaired fasting glucose were more homogeneous than other subgroups. Egger’s regression test did not reveal any publication bias for TC with *p* = 0.050.

#### High-density lipoprotein cholesterol

There was no statistically significant difference in HDL levels between the PA and EH groups (SMD − 0.08 mmol/L; 96%CI (− 0.23, 0.07)). Regarding subgroup analysis, HDL levels were found to be significantly lower in studies with non-Asians, studies with a mean patient age ≥ 50 years, and studies with mean FPG in the impaired-fasting range. Non-significant trends in the same direction were observed in subgroups with predominantly Asians, and patients with a mean age < 50 years and patients with a mean FPG in the normal range. The were no differences in HDL levels between PA and EH patients on BMI and demographic data matching subgroups. There was high heterogeneity among the studies in HDL levels (I^2^ = 87%, *p* < 0.001)). Mean BMI was the only source of heterogeneity. Studies performed predominantly in patients with mean BMI in the non-obese range were more homogeneous than those with mostly obese patients. Egger’s regression test did not reveal any publication bias for HDL with *p* = 0.821.

#### Low-density lipoprotein cholesterol

Patients with PA showed significantly lower levels than those with EH (SMD − 0.17 mmol/L; 95%CI (− 0.27, − 0.08)). For subgroup analysis, significantly lower levels in PA than in EH were reported in studies with predominantly non-obese patients, mean glucose in the normal range and studies with demographic data matching. Non-significant trends in the same direction were found in subgroups of studies with predominantly obese patients, patients with mean glucose in the impaired range and in non-matching demographic data. PA patients in both age groups and of both ethnicity categories showed significantly lower levels of LDL than EH patients. There was moderate heterogeneity among the studies in LDL levels (I^2^ = 65.7%, *p* < 0.001)). Ethnicity and mean FPG were the main sources of heterogeneity. Studies with Asian-predominant patients and normal mean FPG were more homogeneous than other subgroups. Egger’s regression test did not reveal any publication bias for LDL with *p* = 0.311.

### Sensitivity analysis

**Fig. 3:**
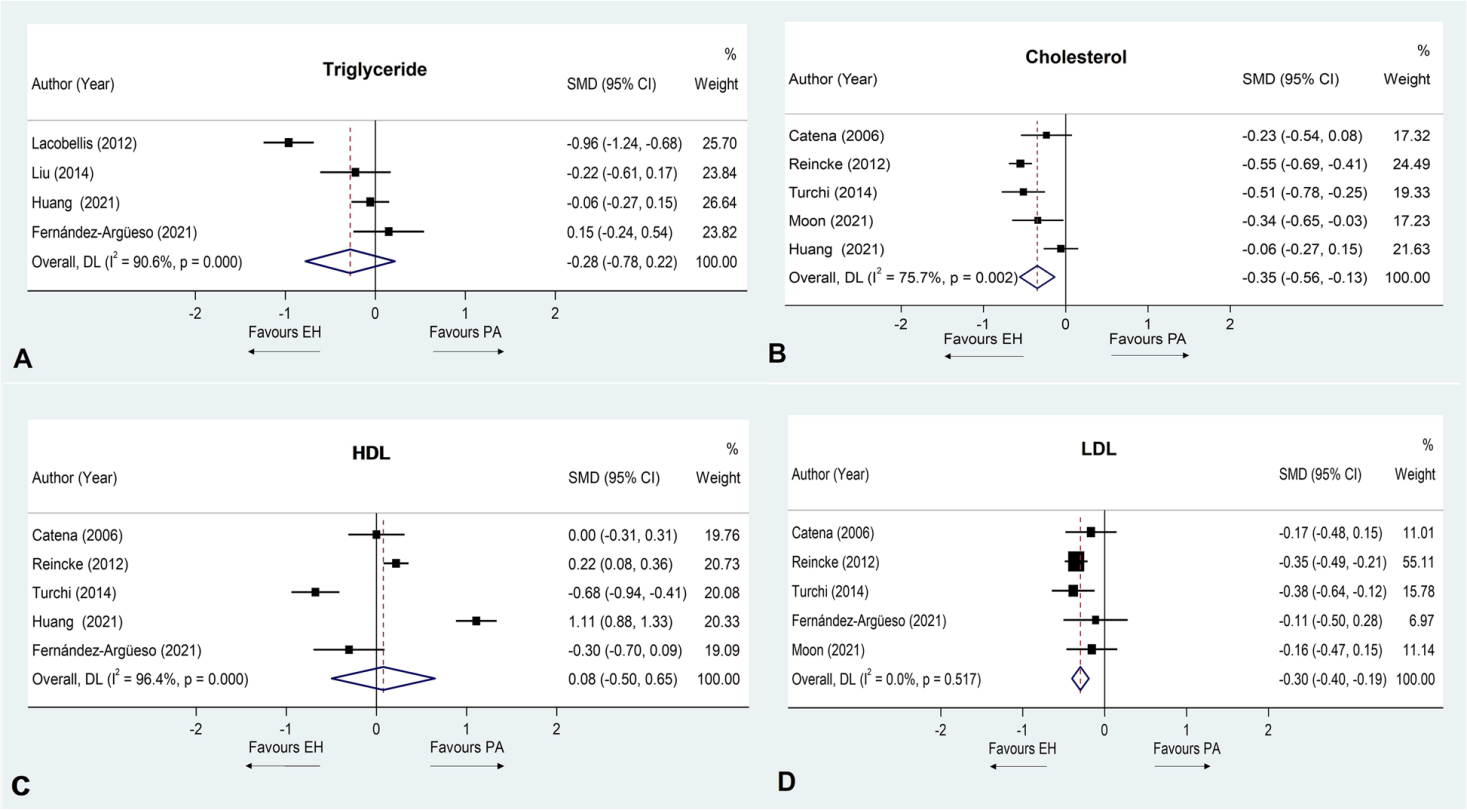
Forest plot of the sensitivity analysis presented by mean difference of triglyceride (**A**), cholesterol (**B**), HDL (**C**) and LDL (**D**) levels between primary aldosteronism and essential hypertension patients

By removing cross-sectional studies from the analysis, results for TC, HDL and LDL remained the same as in the previous analysis. Significantly lower levels of TC and LDL in PA than in EH patients were observed (SMD − 0.35 mmol/L; 95%CI (− 0.56, − 0.13), SMD − 0.30 mmol/L; 95%CI (− 0.40, − 0.19), respectively) and no significant differences of HDL were observed between PA and EH patients (SMD 0.08 mmol/L; 95%CI (− 0.50, 0.65). The results of TG showed no statistically significant difference between the PA and EH groups, which was different from the primary analysis (SMD − 0.28 mmol/L; 95%CI (− 0.78, 0.22)). Regarding heterogeneity among studies, only results for LDL showed an improvement in the heterogeneity. Forest plots of the sensitivity analysis are shown in Fig. 3.

### Certainty of the evidence

The initial assessments for certainty of evidence for TG, TC and LDL SMD values were low due to the observational studies (2+). Considering the moderate to low risk of bias (no rating change), the large heterogeneity in all studies (downgrade one level), indirectness (no rating change), low imprecision and the absence of publication bias (no rating change), the overall level of certainty was very low (1+). For HDL SMD levels, the initial assessment showed low certainty of evidence (2+). There was a high degree of imprecision according to the wide CI with threshold crossing (downgrade one level) and the large heterogeneity in the studies (downgrade one level). Other aspects required no rating change. Therefore, the overall level of certainty was very low for HDL (1+).

## Discussion

This meta-analysis is the first to investigate differences in serum lipid profiles, including TG, TC, HDL and LDL, between PA and EH patients. The study found significant differences in the direction of lower TG, TC and LDL in PA patients than in EH patients. HDL levels were not significantly different between PA and EH patients, but did show a trend toward lower HDL in PA than in EH patients.

It has been demonstrated that dyslipidemia can contribute to an increased risk of atherosclerotic events and cardiovascular complications [53]. In patients with PA, increased risk of atherosclerotic cardio- and cerebrovascular disease has also been observed [54]. The evidence regarding whether dyslipidemia is the cause of these atherosclerotic events in PA is still inconclusive. Evidence of a relationship between plasma aldosterone and alteration of lipid levels in the general population without PA has been reported. However, the association was different to our meta-analysis findings. In patients with metabolic syndrome and without PA, increased plasma aldosterone has been shown to be related to increased TG, LDL and decreased HDL levels [6, 55, 56]. Furthermore, research conducted as part of the Framingham offspring study in participants without metabolic syndrome demonstrated that increased plasma aldosterone levels are linked with an increased TC/HDL ratio but are not associated with HDL levels alone [57]. The explanation underpinning this relationship has been widely discussed but is still inadequately understood. One of the proposed theories is that secretion factors from adipose tissue can stimulate aldosterone secretion, which, in turn, acts on the mineralocorticoid receptor that can mediate adipogenesis [58]. Another hypothesis is that high aldosterone levels can provoke acute renal failure which, as a consequence, can then alter LDL and HDL production [59].

Unlike the situation in the general population, our meta-analysis of lipid levels, including TG, TC and LDL, in PA patients showed significantly lower levels than in EH patients. Lower plasma TG in PA than in EH patients could be the result of glomerular hyperfiltration caused by hypertension and to the increased plasma volume which is commonly observed in PA patients [60]. Increased levels of TG can be observed in chronic renal failure patients, a result of low activity of lipoprotein lipase (LPL) which facilitates clearance of TG and reduction of TG levels [61]. Thus, an increase in the estimated glomerular filtration rate (eGFR) in PA patients can contribute to increases in LPL activity which results in increased TG clearance and, finally, decreased TG levels. An interesting study supporting this hypothesis reported that successfully treated PA patients showed worsening TG levels despite improvement in blood pressure and glucose levels which could be explained by the decline in glomerular filtration after adrenalectomy [8]. Currently, the mechanism responsible for lower TC and LDL in PA patients than in EH patients is still uncertain. Circulating lipoproteins are the source of precursors of adrenal steroidogenesis, including aldosterone production. However, it cannot be directly inferred that increased production of aldosterone in PA can cause the depletion of TC and LDL and lead to levels reduction [62]. Another possible explanation could relate to liver X receptors (LXR) which are involved in cholesterol metabolism. It is known that LXR stimulation can increase renin and aldosterone accumulation, but whether the existence of a backward causation effect of aldosterone can stimulate LXR is still unclear [63]. Moreover, there were other factors which could affect lipid profiles, including genetic mutations, diet, physical activity, obesity, smoking, diabetes mellitus, hypothyroidism, liver diseases, renal function, and some medications. Regarding medications, thiazide type diuretics, which is an anti-hypertensive medication commonly used in EH, could lead to increased TG, TC, and LDL, while mineralocorticoid receptor blockers, the drug of choice for PA, have no effect on lipid profiles. This could be one of the explanations for the different lipid changes between PA and EH [64]. Nevertheless, these factors, which can interfere with lipid levels, were not reported in the included studies. Future studies to address the link between PA and these factors are warranted.

Significantly lower levels of TG, TC and LDL in PA than in EH patients were an unexpected finding taking into consideration the relatively high incidence of atherosclerotic events, metabolic syndrome and insulin resistance in PA patients [37, 65]. These findings may be used as a guide for the clinicians to differentiate between PA and EH, in which, those with low levels of TG, TC and LDL, PA should be suspected. Nevertheless, future research regarding this issue should be warranted.

As mentioned above, increased aldosterone levels can cause oxidative stress and pro-inflammatory effects on vascular walls; this is in line with the recent observation that elevated secretion of aldosterone increases oxidized small dense LDL, as indirectly measured by the TG/HDL-C ratio [66]. It is therefore likely that patients with primary aldosteronism may have alterations in both the quantity and the quality of LDL, with reduced LDL concentrations but with increased levels of atherogenic small dense LDL [67]; these particles are strongly associated with atherosclerosis formation and progression [68]. Moreover, it is known that elevated aldosterone levels is associated with elevated levels of pro-inflammatory adipokines such as resistin [36], which are also strongly associated with atherogenic small dense LDL [69] and therefore potentially enhancing the atherosclerotic process.

However, due to the moderate to high heterogeneity among the studies, the results of this review should be interpreted cautiously. Concerning the heterogeneity issue, confounders which could potentially interfere with the levels of lipid profiles were addressed by subgroup analyses. In terms of ethnicity, non-Asian predominant studies reported lower levels of TG, TC and LDL in PA than in EH patients. Asian populations demonstrated an increased trend of age-standardized non-HDL cholesterol and cholesterol levels to a greater extent than did Caucasians [70]. Available data showed that the maximum doses of statins used in Western countries were higher than in Asian countries [71]. This may be the result of differences in lipid profiles between the ethnic groups. However, the direct relationship between ethnicity and levels of aldosterone and lipids has not yet been clearly elucidated. Data on dosages and percentages of statin use was not provided in the studies included in this meta-analysis.

Levels of TC and HDL were observed to be lower in PA than in EH, particularly in studies with non-obese patients predominant. We surmised that non-obese patients with PA may have a higher risk of diabetes than EH patients due to increased insulin resistance from high aldosterone [72]. This is consistent with previous suggestions on a patho-physiological link between diabetic lipoproteins and adrenal aldosterone synthesis [73]. Diabetic patients seem to be more likely to have been prescribed statins, resulting in lower cholesterol levels. However, data on diabetes prevalence and statin use was not provided in the included studies. TC and LDL levels were observed to be lower in PA than in EH patients only in studies with mean FPG in the impaired glucose level range. As noted above, patients diagnosed with impaired fasting glucose were more likely to be prescribed statins than those with normal glucose. Levels of TC were significantly lower in PA than in EH patients only in those studies that have been matched for demographic data. This could indicate that after removing demographic confounders using case-control matching, the remaining difference in lipid levels between PA and EH patients was real.

A sensitivity analysis by removing cross-sectional studies was performed, and only cohort or case-control studies were analyzed. The reason behind this sensitivity analysis is that a cross-sectional study can determine the prevalence or associations rather than causality, whereas a cohort or case-control study can establish the timing and directionality of events. The results remained almost the same as in the previous analysis, except for that of TG. However, since the main objectives of most of the cohort and case-control studies were not aimed at finding the differences in lipid profiles between PA and EH patients, the type of study design may not play a major role in the outcomes.

One of the strengths of this first meta-analysis to address the difference in lipid parameters between PA and EH patients is that only studies which had clear criteria to diagnose PA were selected. Another strength is that subgroup analysis was performed to identify the effects of potential confounders and to identify causes of heterogeneity.

There are, however, several limitations that needed to be acknowledged. The main limitation is the high level of heterogeneity among the included studies, even though subgroup analyses were conducted to investigate the sources and impacts of the heterogeneity. Second, the primary objectives of most of the studies did not aim to find the differences in lipid profiles between PA and EH patients. Additionally, most of the lipid data used in the meta-analyses were obtained from baseline characteristics. That may have affected outcomes as some confounders, e.g., sex, age, BMI and blood pressure, were not matched prior to the analysis. However, in the analyses of subgroups established by demographic data matching, TG, HDL and LDL levels were still significantly lower in PA than in EH patients. Third, only two of the studies excluded patients who had used statins prior to the analyses which could have affected lipid measurements. Nevertheless, subgroup analysis by statin use showed similar TG result between two subgroups of no statin group and statin use/unknown status of use group. Additionally, lifestyle, activity level, diet and genetics may possibly affect lipid profiles, but most of the studies did not provide information regarding these factors. Also, the measurement of lipid profiles, especially LDL levels, can be performed by either a direct measurement method or by using various equations. However, the method employed to measure LDL was not reported in the included studies. This can lead to different results for LDL among the studies [74].

## Conclusion

Compared to EH patients, PA patients had unexpectedly and significantly lower levels of lipid parameters including TG, TC and LDL. This indicates that increased atherosclerotic cardiovascular events in PA patients are not a result of differences in lipid levels. Further research is required to address the mechanisms involved in lipid metabolism in PA.

## Supplementary Information

**Additional file 1: Table S1.** Keywords of articles searching. **Table S2.1.** Risk of bias assessed by Joanna Briggs Institute (JBI) Critical Appraisal Tools for cross-sectional study. **Table S2.2.** Risk of bias assessed by Joanna Briggs Institute (JBI) Critical Appraisal Tools for case-control study. **Table S2.3.** Risk of bias assessed by Joanna Briggs Institute (JBI) Critical Appraisal Tools for cohort study. **Figure S1.** Subgroup analysis by age group. **Figure S2.** Subgroup analysis by age group. **Figure S3.** Subgroup analysis by BMI. **Figure S4.** Subgroup analysis by blood glucose. **Figure S5.** Subgroup analysis by demographic data matching. **Figure S6.** Subgroup analysis by statin use. **Figure S7.** Funnel plots.

## Data Availability

The datasets used and/or analysed during the current study are available at https://www.dropbox.com/s/f1bz1g0tsx0ezjg/raw%20data%20new1.xlsx?dl=0

## Abbreviations

BMI: Body mass index
CI: Confidence interval
EH: Essential hypertension
FPG: Fasting plasma glucose
HDL: High density lipoprotein
LDL: Low density lipoprotein
PA: primary aldosteronism
SMD: Standardized mean difference
TC: Total cholesterol
TG: Triglyceride
