# Supplementary material for: The differences of serum lipid profiles between primary aldosteronism and essential hypertension: a meta-analysis and systematic review
Source: BMC Endocr Disord. 2022 Aug 31;22:217. doi: 10.1186/s12902-022-01135-y (PMC9429522; doi:10.1186/s12902-022-01135-y)

**Supplementary Appendix**

**Table S1.** Keywords of articles searching

**Table S2.1.** Risk of bias assessed by Joanna Briggs Institute (JBI) Critical Appraisal Tools for cross-sectional study

**Table S2.2.** Risk of bias assessed by Joanna Briggs Institute (JBI) Critical Appraisal Tools for case-control study

**Table S2.3.** Risk of bias assessed by Joanna Briggs Institute (JBI) Critical Appraisal Tools for cohort study

**Figure S1.** Subgroup analysis by age group

**Figure S2.** Subgroup analysis by age group

**Figure S3.** Subgroup analysis by BMI

**Figure S4.** Subgroup analysis by blood glucose

**Figure S5.** Subgroup analysis by demographic data matching

**Figure S6.** Subgroup analysis by statin use

**Figure S7.** Funnel plots

**Table S1.** Keywords for articles searches

| **Keywords** | **Number of articles** |
| --- | --- |
| ***Pubmed*** | 3,399 |
| ("Hyperaldosteronism"[Mesh] OR aldosteronism OR primary aldosteronism) AND (lipid OR "Lipids"[Mesh] OR "Cholesterol"[Mesh] OR "Cholesterol, LDL"[Mesh] OR "Cholesterol, HDL"[Mesh] OR "Triglycerides"[Mesh] OR "Metabolic Syndrome"[Mesh] OR “apolipoprotein A” OR “apolipoprotein B′′ OR “docosahexanoic acid” OR “omega 3 fatty acid” OR “omega 6 fatty acid” OR “polyunsaturated acid”) |  |
| ***Embase*** | 951 |
| ('metabolic syndrome x'/exp OR 'lipid'/exp OR 'cholesterol'/exp OR 'low density lipoprotein cholesterol'/exp OR 'fatty acid'/exp OR 'high density lipoprotein cholesterol'/exp OR 'apolipoprotein A'/exp OR 'apolipoprotein B'/exp OR 'docosahexanoic acid'/exp OR 'omega 3 fatty acid'/exp OR 'omega 6 fatty acid'/exp OR 'polyunsaturated fatty acid'/exp) AND 'hyperaldosteronism'/exp |  |
| ***Scopus*** | 1,775 |
| ("metabolic syndrome" OR "metabolic" OR "cholesterol" OR "triglyceride" OR "low density lipoprotein" OR "high density lipoprotein" OR "lipid" OR "profile" OR “apolipoprotein A” OR “apolipoprotein B” OR “docosahexanoic acid” OR “omega 3 fatty acid” OR “omega 6 fatty acid” OR “polyunsaturated acid”) AND ( "hyperaldosteronism" OR "aldosteronism" OR "primary aldosteronism") |  |

**Search outputs link**: https://www.dropbox.com/sh/364y2nxcizi5x95/AACke2nZJj-UfzKFnX8zItAra?dl=0

**Table S2.1.** Risk of bias assessed using Joanna Briggs Institute (JBI) Critical Appraisal Tools for cross-sectional study

| **Question** | **Strauch**  **2006** | **Fallo**  **2006** | **Ronconi**  **2009** | **Matrozova**  **2009** | **Somloova**  **2010** | **Fallo**  **2010** | **Stehr**  **2010** | **Iacobellis**  **2012** | **Savard**  **2013** | **Prejbisz**  **2013** | **Liu**  **2014** | **Cloudhary**  **2015** | **Yang**  **2016** |
| --- | --- | --- | --- | --- | --- | --- | --- | --- | --- | --- | --- | --- | --- |
| Were the criteria for inclusion in the sample clearly defined? | x | / | / | / | / | / | / | / | / | / | / | / | / |
| Were the study subjects and the setting described in detail? | / | / | / | / | / | / | / | / | / | / | / | / | / |
| Was the exposure measured in a valid and reliable way? | / | / | / | / | / | / | / | / | / | / | / | / | / |
| Were objective, standard criteria used for measurement of the condition? | / | / | / | / | / | / | / | / | / | / | / | x | / |
| Were confounding factors identified? | / | / | / | / | / | / | / | / | / | X | / | / | / |
| Were strategies to deal with confounding factors stated? | x | / | / | / | / | / | / | / | / | X | / | / | / |
| Were the outcomes measured in a valid and reliable way? | N/A | / | / | / | / | N/A | N/A | / | N/A | / | N/A | N/A | N/A |
| Was appropriate statistical analysis used? | x | / | / | / | / | / | / | / | / | X | / | / | / |
| Total | 4 | 8 | 8 | 8 | 8 | 7 | 7 | 8 | 7 | 5 | 7 | 6 | 7 |
| Risk of bias | Moderate | Low | Low | Low | Low | Low | Low | Low | Low | Moderate | Low | Moderate | Low |

| **Question** | **Watanabe**  **2016** | **Monticone**  **2017** | **Berends**  **2018** | **Li**  **2018** | **Er**  **2019** | **Vujacik**  **2020** | **Manosroi**  **2020** | **Zhang 2020** | **Hu**  **2020** | **Caprino**  **2020** | **Sang 2021** | **Moon**  **2021** |
| --- | --- | --- | --- | --- | --- | --- | --- | --- | --- | --- | --- | --- |
| Were the criteria for inclusion in the sample clearly defined? | / | / | / | / | / | / | / | / | / | / | / | / |
| Were the study subjects and the setting described in detail? | / | / | / | / | / | / | / | / | / | / | / | / |
| Was the exposure measured in a valid and reliable way? | / | / | / | / | / | / | / | / | / | / | / | / |
| Were objective, standard criteria used for measurement of the condition? | / | / | / | / | / | / | / | / | / | / | / | / |
| Were confounding factors identified? | / | / | / | / | / | / | / | / | / | / | X | / |
| Were strategies to deal with confounding factors stated? | / | / | / | / | / | / | / | / | / | / | X | / |
| Were the outcomes measured in a valid and reliable way? | N/A | / | / | N/A | N/A | / | N/A | N/A | N/A | N/A | N/A | / |
| Was appropriate statistical analysis used? | / | / | / | / | / | X | / | / | / | / | X | / |
| Total | 7 | 8 | 8 | 7 | 7 | 7 | 7 | 7 | 7 | 7 | 4 | 8 |
| Risk of bias | Low | Low | Low | Low | Low | Low | Low | Low | Low | Low | Moderate | Low |

Low risk: scores ≥7

Moderate risk: scores 4-6

High risk: scores <4

**Table S2.2.** Risk of bias assessed using Joanna Briggs Institute (JBI) Critical Appraisal Tools for case-control study

| **Question** | **Reincke**  **2012** | **Fernández-Argüeso**  **2021** | **Huang**  **2021** |
| --- | --- | --- | --- |
| Were the groups comparable other than the presence of disease in cases or the absence of disease in controls? | / | / | / |
| Were cases and controls matched appropriately? | / | / | / |
| Were the same criteria used for identification of cases and controls? | / | / | / |
| Was exposure measured in a standard, valid and reliable way? | / | / | / |
| Was exposure measured in the same way for cases and controls? | / | / | / |
| Were confounding factors identified? | / | / | / |
| Were strategies to deal with confounding factors stated? | / | / | / |
| Were outcomes assessed in a standard, valid and reliable way for cases and controls? | N/A | N/A | N/A |
| Was the exposure period of interest long enough to be meaningful? | N/A | N/A | N/A |
| Was appropriate statistical analysis used? | / | / | / |
| Total | 8 | 8 | 8 |
| Risk of bias | Low | Low | Low |

High risk: ≤49% of “yes” scores

Moderate risk: 50 to 69% of “yes” scores

Low risk: more than 70% of “yes” scores

**Table S2.3.** Risk of bias assessed by Joanna Briggs Institute (JBI) Critical Appraisal Tools for cohort study

| **Question** | **Catena**  **2006** | **Turchi**  **2014** |
| --- | --- | --- |
| Were the two groups similar and recruited from the same population? | / | / |
| Were the exposures measured similarly to assign people to both exposed and unexposed groups? | / | / |
| Was the exposure measured in a valid and reliable way? | / | / |
| Were confounding factors identified? | / | / |
| Were strategies to deal with confounding factors stated? | / | / |
| Were the groups/participants free of the outcome at the start of the study (or at the moment of exposure)? | N/A | N/A |
| Were the outcomes measured in a valid and reliable way? | N/A | / |
| Was the follow up time reported and sufficient to be long enough for outcomes to occur? | N/A | N/A |
| Was follow up complete, and if not, were the reasons to loss to follow up described and explored? | N/A | N/A |
| Were strategies to address incomplete follow up utilized? | N/A | N/A |
| Was appropriate statistical analysis used? | / | / |
| Total | 6 | 7 |
| Risk of bias | Moderate | Moderate |

High risk: ≤49% of “yes” scores

Moderate risk: 50 to 69% of “yes” scores

Low risk: more than 70% of “yes” scores

**Figure S1.** Subgroup analysis by age group showing Forest plots of the mean difference in triglyceride (A), cholesterol (B), HDL (C) and LDL (D) levels between primary aldosteronism and essential hypertension patients

**
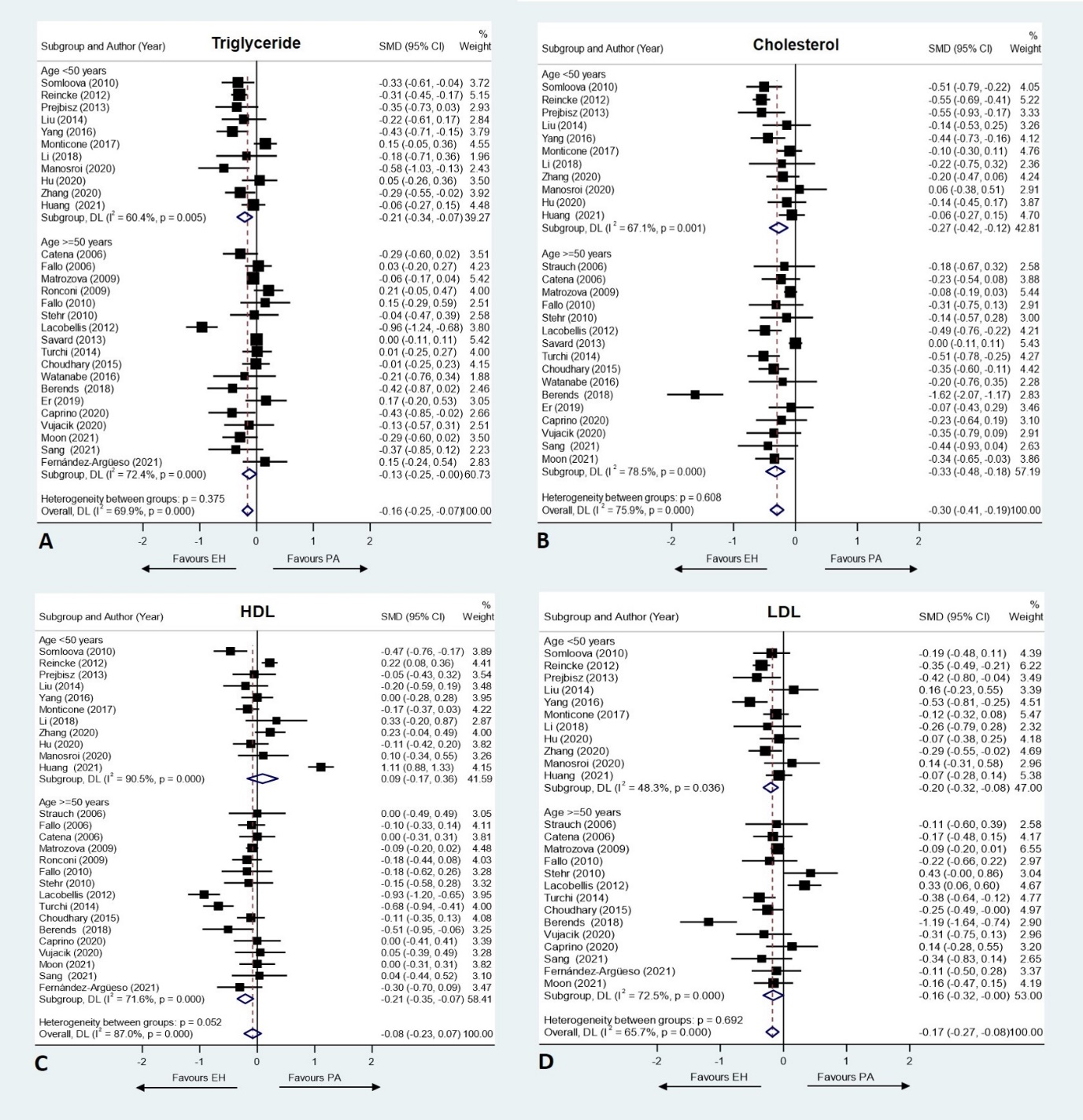
**

**Figure S2.** Subgroup analysis by ethnicity showing Forest plots of the mean difference in triglyceride (A), cholesterol (B), HDL (C) and LDL (D) levels between primary aldosteronism and essential hypertension patients


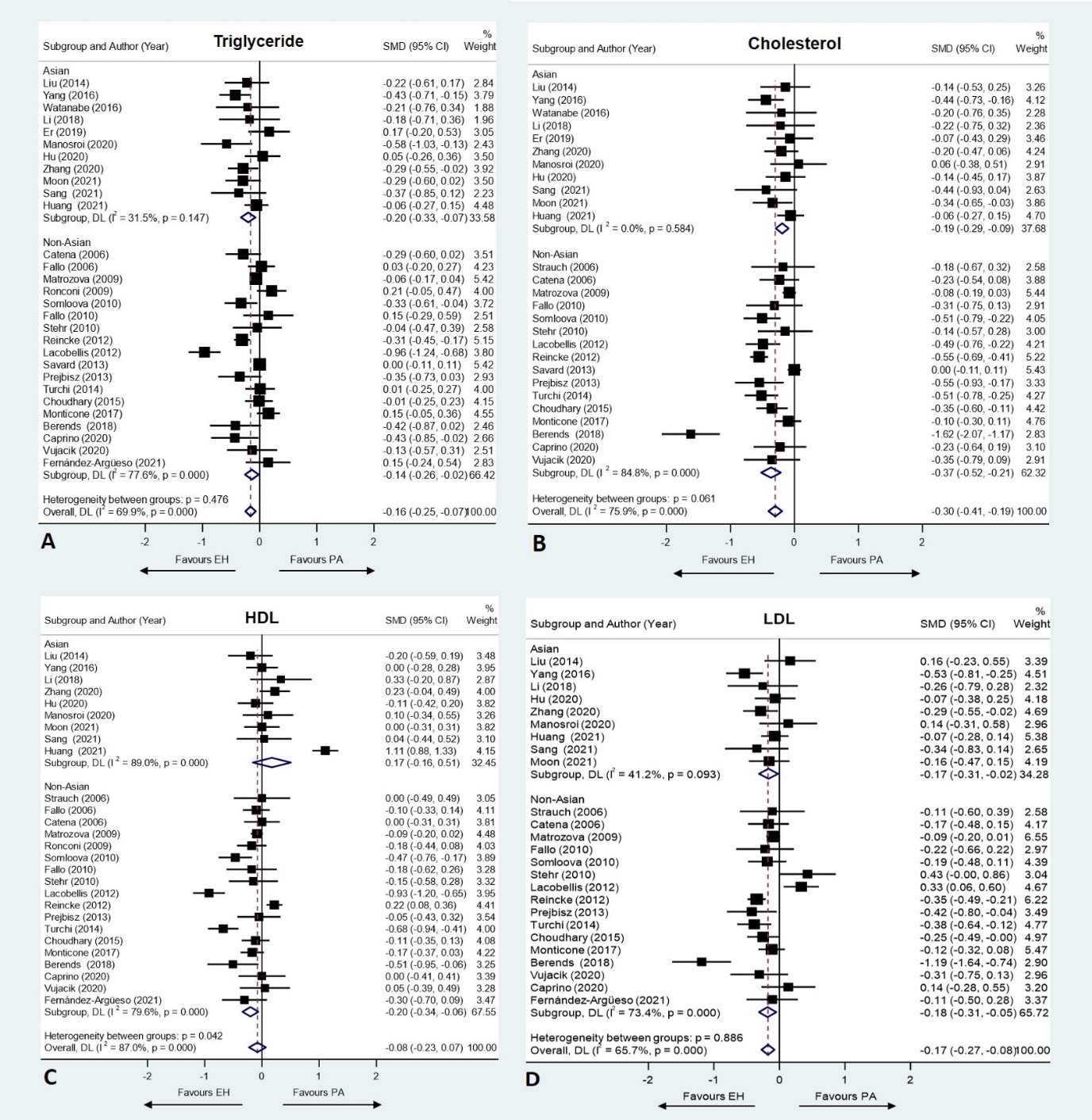


**Figure S3.** Subgroup analysis by BMI showing Forest plot of the mean difference in triglyceride (A), cholesterol (B), HDL (C) and LDL (D) levels between primary aldosteronism and essential hypertension patients

**
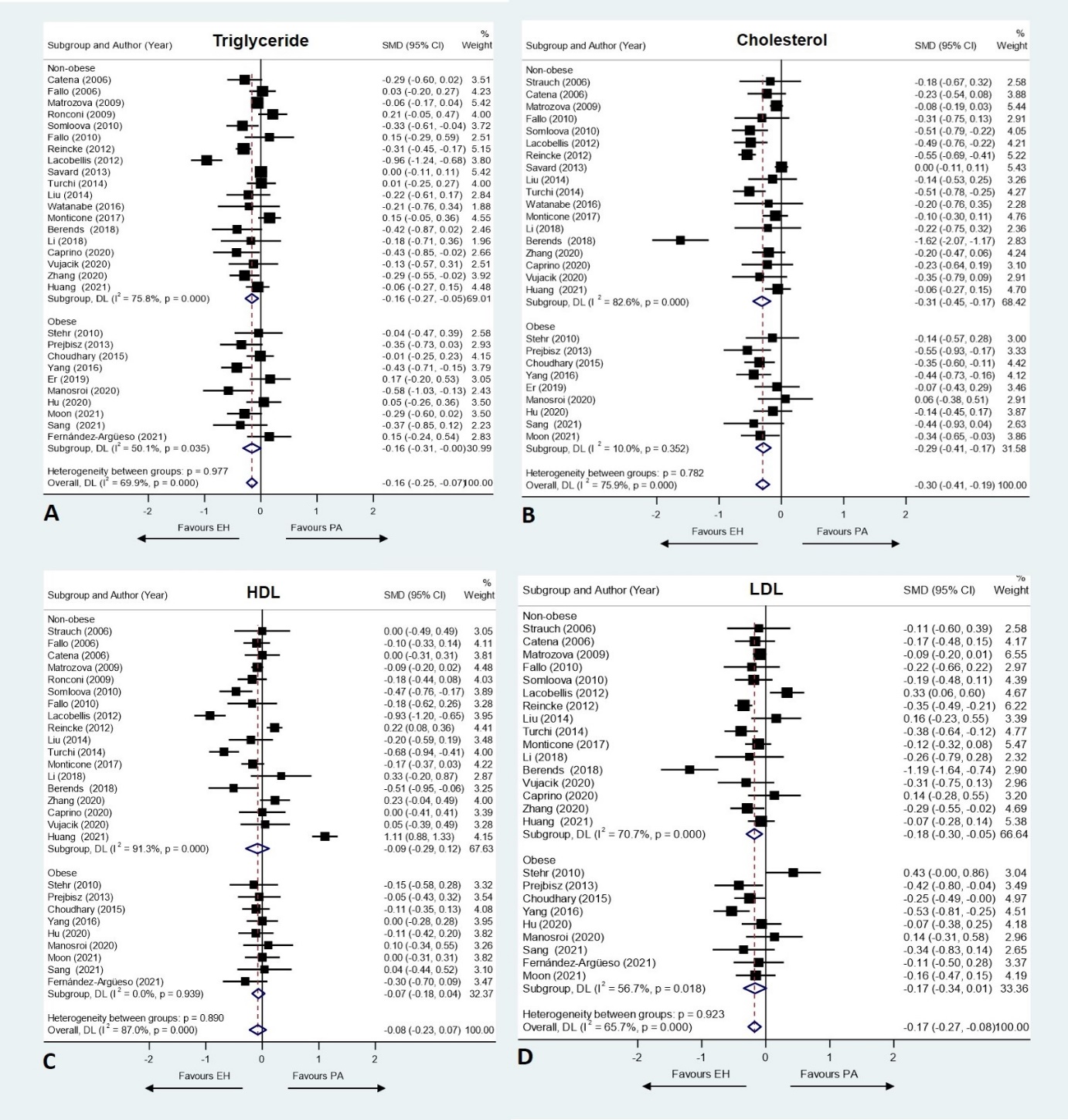
**

**Figure S4.** Subgroup analysis by blood glucose showing Forest plots of the mean difference in triglyceride (A), cholesterol (B), HDL (C) and LDL (D) levels between primary aldosteronism and essential hypertension patients

**
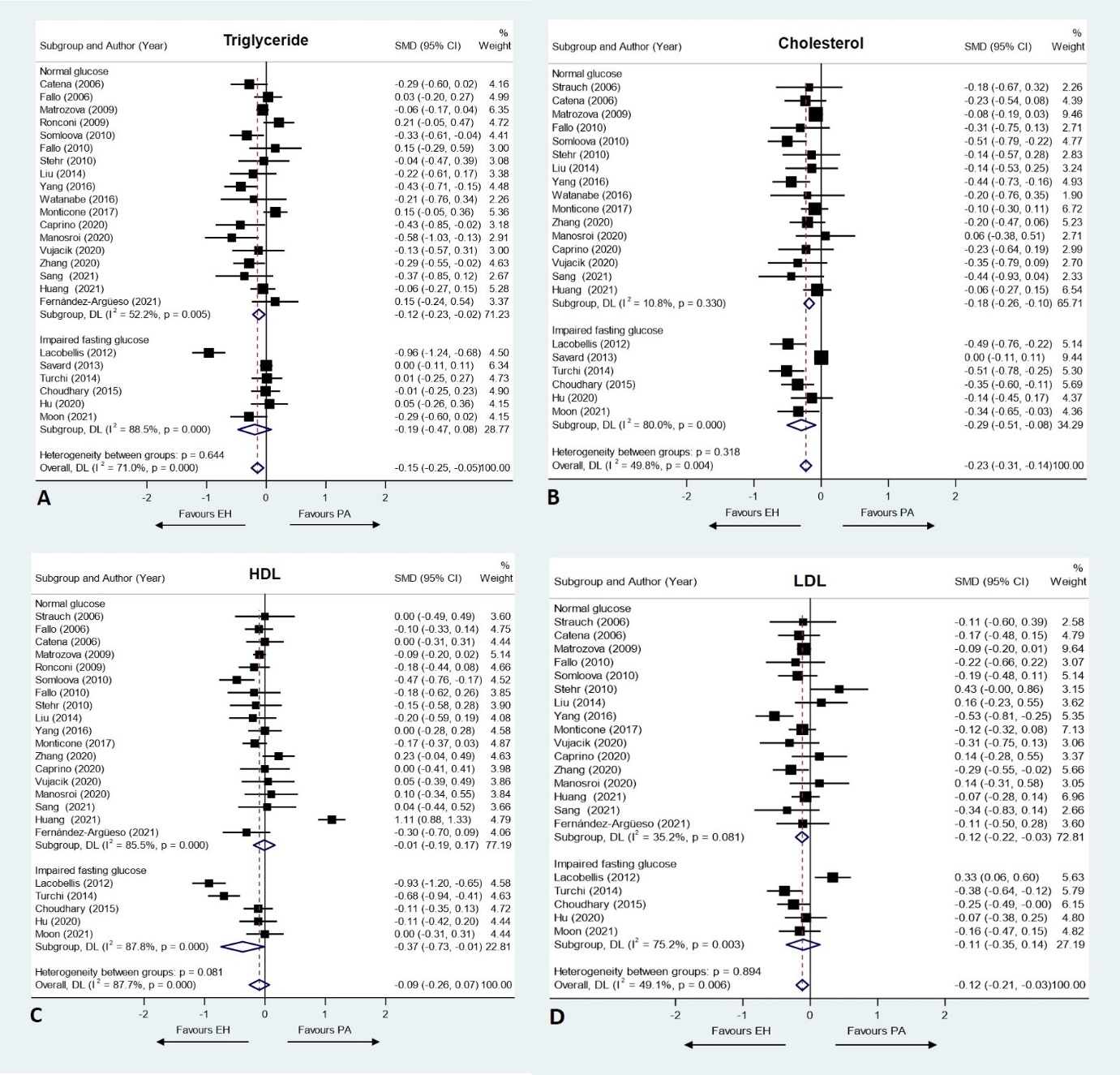
**

**Figure S5.** Subgroup analysis by demographic data matching showing Forest plots of the mean difference in triglyceride (A), cholesterol (B), HDL (C) and LDL (D) levels between primary aldosteronism and essential hypertension patients

**
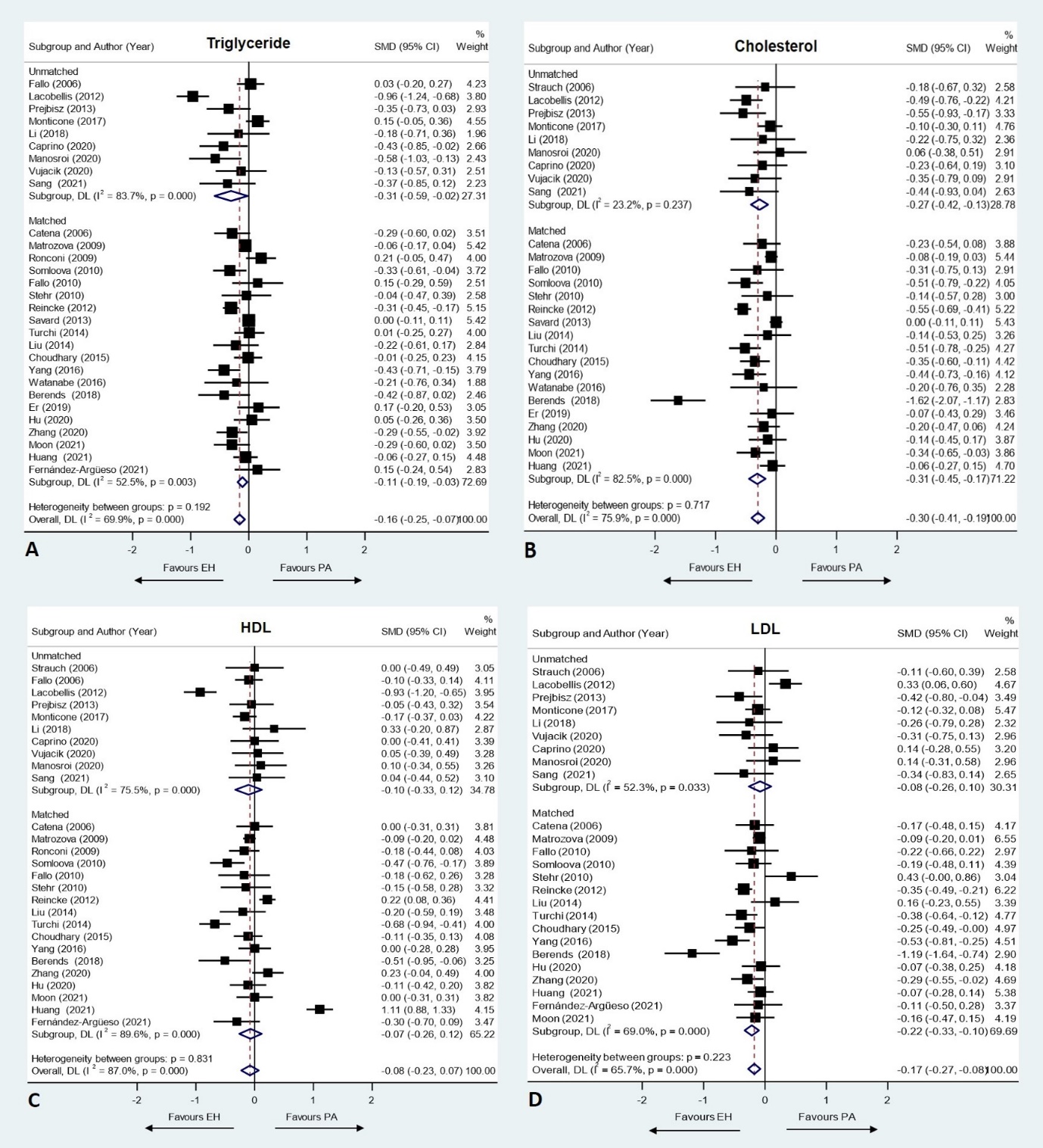
**

**Figure S6.** Subgroup analysis by statin use showing Forest plots of the mean difference in triglyceride between primary aldosteronism and essential hypertension patients

**
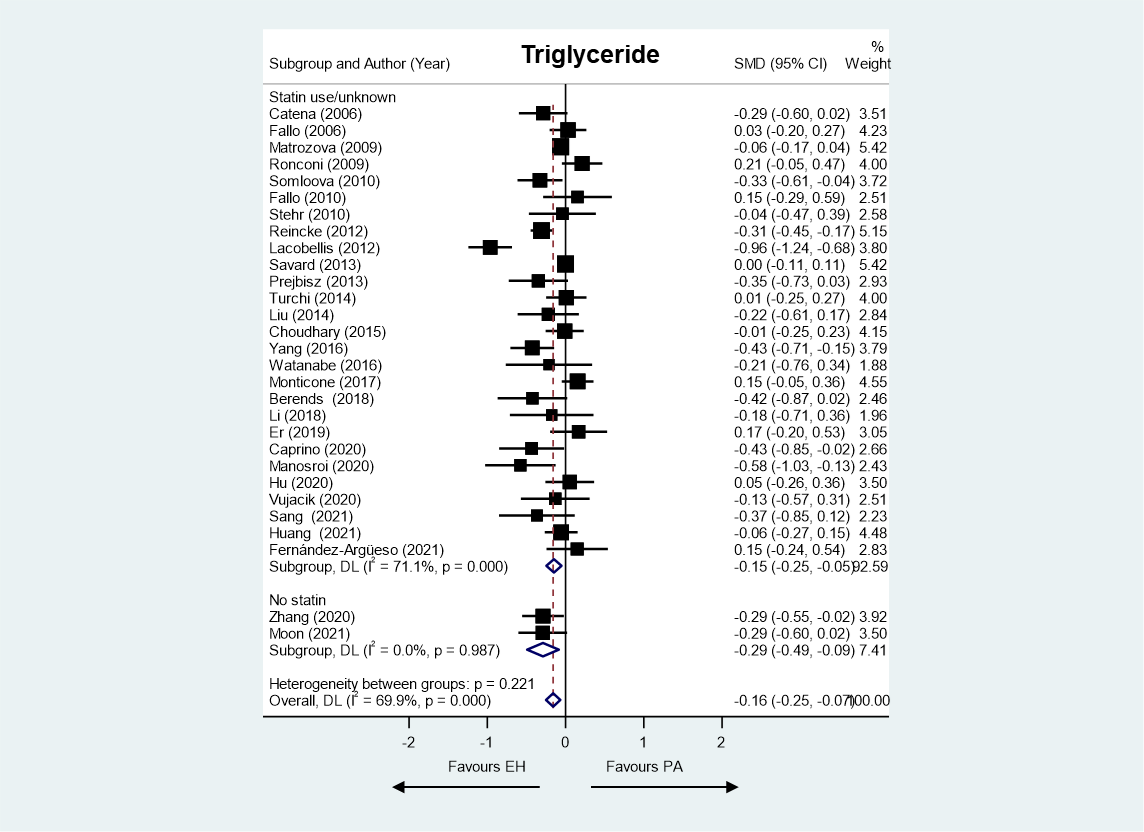
**

**Figure S7.** Funnel plots


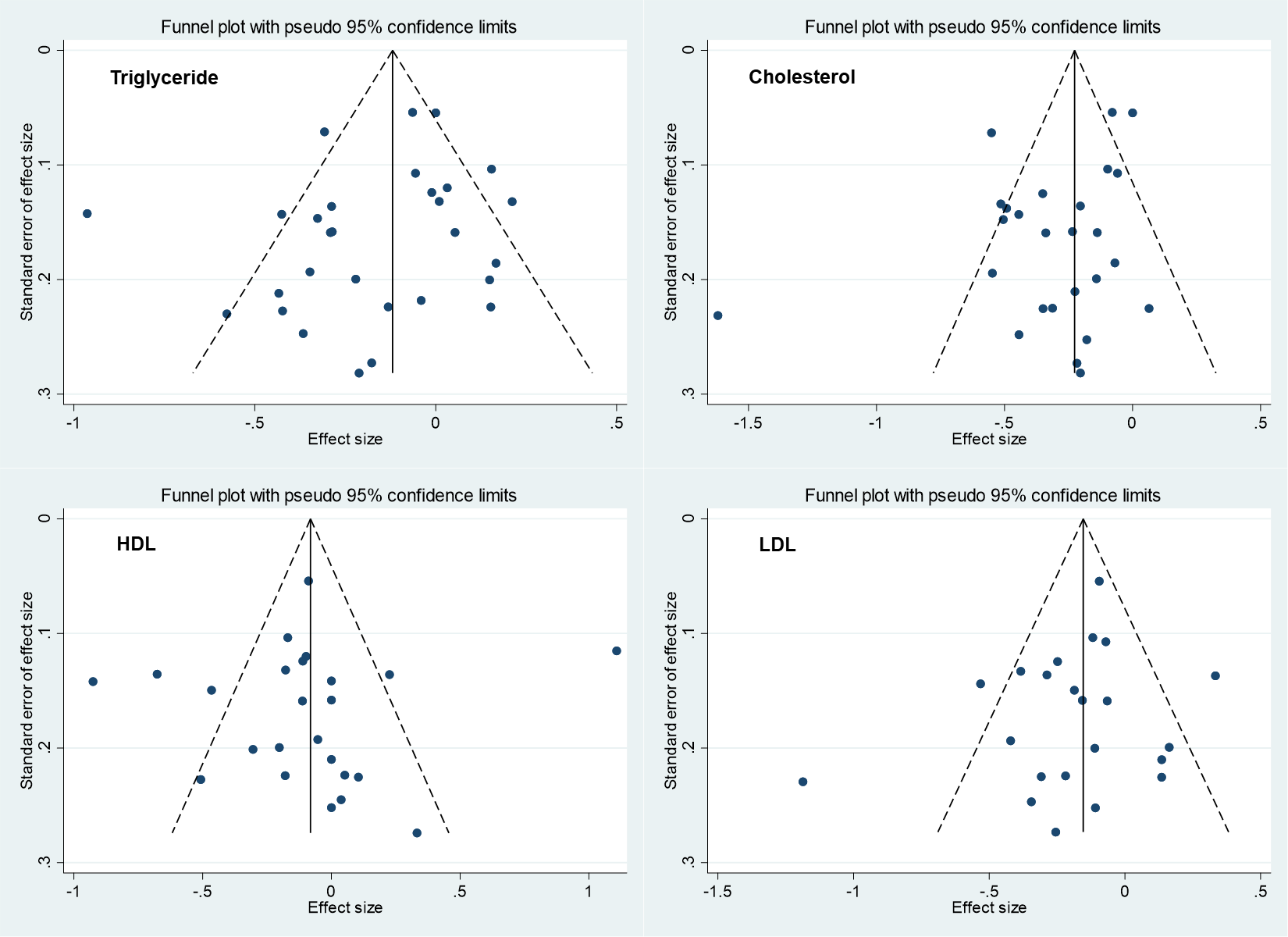

Supplement: Supplementary file 1 — Additional file 1: Table S1. Keywords of articles searching. Table S2.1. Risk of bias assessed by Joanna Briggs Institute (JBI) Critical Appraisal Tools for cross-sectional study. Table S2.2. Risk of bias assessed by Joanna Briggs Institute (JBI) Critical Appraisal Tools for case-control study. Table S2.3. Risk of bias assessed by Joanna Briggs Institute (JBI) Critical Appraisal Tools for cohort study. Figure S1. Subgroup analysis by age group. Figure S2. Subgroup analysis by age group. Figure S3. Subgroup analysis by BMI. Figure S4. Subgroup analysis by blood glucose. Figure S5. Subgroup analysis by demographic data matching. Figure S6. Subgroup analysis by statin use. Figure S7. Funnel plots. [file 12902_2022_1135_MOESM1_ESM.docx]
